# Gestational Pseudoangiomatous Stromal Hyperplasia Presenting as Gigantomastia: A Case Report of a Rare Breast Entity with Clinical Recommendations by a Multidisciplinary Team

**DOI:** 10.1155/2023/9279934

**Published:** 2023-07-10

**Authors:** S. Jennifer Wang, Shivi Maheswaran, Rosemary Reiss, Leah H. Portnow, Jane Brock, Lara Novak, Jessica Erdmann-Sager, Thanh U. Barbie

**Affiliations:** ^1^Division of Surgical Oncology, Department of Surgery, Brigham and Women's Hospital, Boston, MA, USA; ^2^Division of Breast Surgery, Department of Surgery, Brigham and Women's Hospital, Boston, MA, USA; ^3^Division of Maternal-Fetal Medicine, Department of Obstetrics and Gynecology, Brigham and Women's Hospital, Boston, MA, USA; ^4^Division of Breast Imaging, Department of Radiology, Brigham and Women's Hospital, Boston, MA, USA; ^5^Department of Pathology, Brigham and Women's Hospital, Boston, MA, USA; ^6^Breast Oncology Program, Dana-Farber Brigham Cancer Center, Boston, MA, USA; ^7^Division of Plastic Surgery, Department of Surgery, Brigham and Women's Hospital, Boston, MA, USA

## Abstract

**Introduction:**

Pseudoangiomatous stromal hyperplasia (PASH) presenting as gigantomastia is rare in pregnancy but can result in severe clinical consequences for both mother and fetus. *Case Presentation*. A 43-year-old female with a history of biopsy-proven bilateral PASH presented at 22 3/7 weeks gestation with massive bilateral breast enlargement that was symptomatic. After multidisciplinary care, she underwent bilateral mastectomies and delivered at term with no additional complications.

**Conclusion:**

Pregnant women who undergo mastectomies for PASH-induced gigantomastia during their second trimesters will likely recover quickly, and fetal risks are low. Given the rarity of this breast entity, management guidelines are sparse. Our case report is an effort to comprehensively review this condition and share the clinical recommendations made by our institution's multidisciplinary team.

## 1. Introduction

Pseudoangiomatous stromal hyperplasia (PASH) is a benign breast disease appearing in pre- or perimenopausal women. When PASH presents as a symptomatic firm nodule or as breast hypertrophy, the standard of care is conservative surgical excision of PASH-affected tissue [1]. Rarely, severe diffuse PASH results in massive breast hypertrophy [2]. During pregnancy, severe diffuse PASH is worsened by concurrent gestational breast growth [3]. This combination poses risks to both the mother and fetus. Although some case reports describe gestational PASH, management recommendations are sparse. This case report highlights the clinical management of this disease from a multidisciplinary team approach.

## 2. Case Presentation

A 43-year-old primigravida at 22 3/7 weeks presented to our department with massive bilateral breast enlargement. The patient reported progressively worsening shortness of breath and diffuse body pain, which limited her mobility.

### 2.1. Clinical History

**Figure 1:**
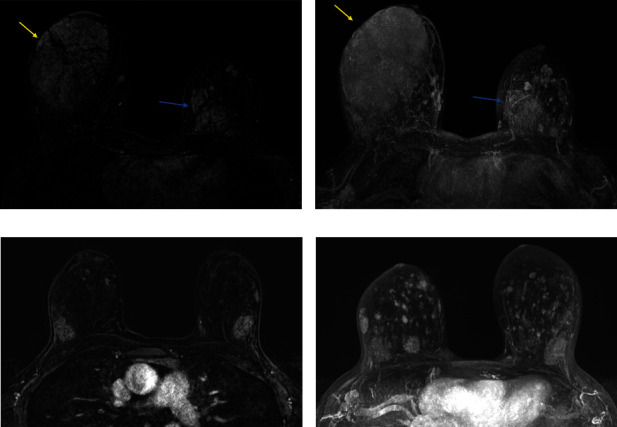
A 39-year-old woman with symptomatic PASH. Contrast-enhanced axial MRI (a) and maximum intensity projection (MIP) (b). Images show a right outer central breast 9 : 00 anterior to a middle depth 10.1 cm heterogeneously enhancing oval mass with circumscribed margins and interspersed areas of fat (arrow), biopsy-proven PASH. The left breast also has a lower inner quadrant 7 : 00 posterior depth 4.1 cm heterogeneously enhancing oval mass with circumscribed margins (thin arrow), also later found to be biopsy-proven PASH. Approximately two years later, after excision of these two masses and treatment with a course of tamoxifen, the patient developed diffuse PASH in both breasts as seen on contrast-enhanced axial MRI (c) and MIP (d) images.

The patient had been diagnosed 4 years earlier with biopsy-proven PASH in a right breast lump that was found when she presented for infertility treatment. The patient thereafter tried to conceive with clomiphene and intrauterine insemination. She then reported marked growth of the right PASH and numbness of the overlying skin that was so symptomatic that she discontinued clomiphene and started a trial of tamoxifen for 3 months. Breast magnetic resonance imaging (MRI) showed bilateral heterogeneously enhancing oval masses with circumscribed margins, biopsies of which confirmed hypertrophic focal right PASH, and new left onset PASH at the time of symptom onset (Figures 1(a) and 1(b)). During this interim, her breast hypertrophy stabilized but did not regress. Given her desire to recommence fertility treatments, she pursued bilateral extensive excisions with oncoplastic closure.

Within 3 months of restarting clomiphene, she again reported symptomatic PASH with skin discomfort. Given the rapid recurrence, definitive surgery with mastectomy was offered but she declined. Six months later, after her first cycle of in vitro fertilization (IVF), she had increased bilateral breast enlargement but continued with expectant management of PASH. After a year of attempting to conceive by IVF, MRI showed that the PASH had become more generalized and diffuse (Figures 1(c) and 1(d)), so focal excisional biopsies were no longer an option. Five months before the embryo transfer that led to her successful conception, the patient reported worsening skin symptoms and more rapid breast enlargement (left greater than right) and underwent a left reduction mammoplasty. A mastectomy procedure was offered but the patient declined.

### 2.2. Presentation during Pregnancy

**Figure 2:**
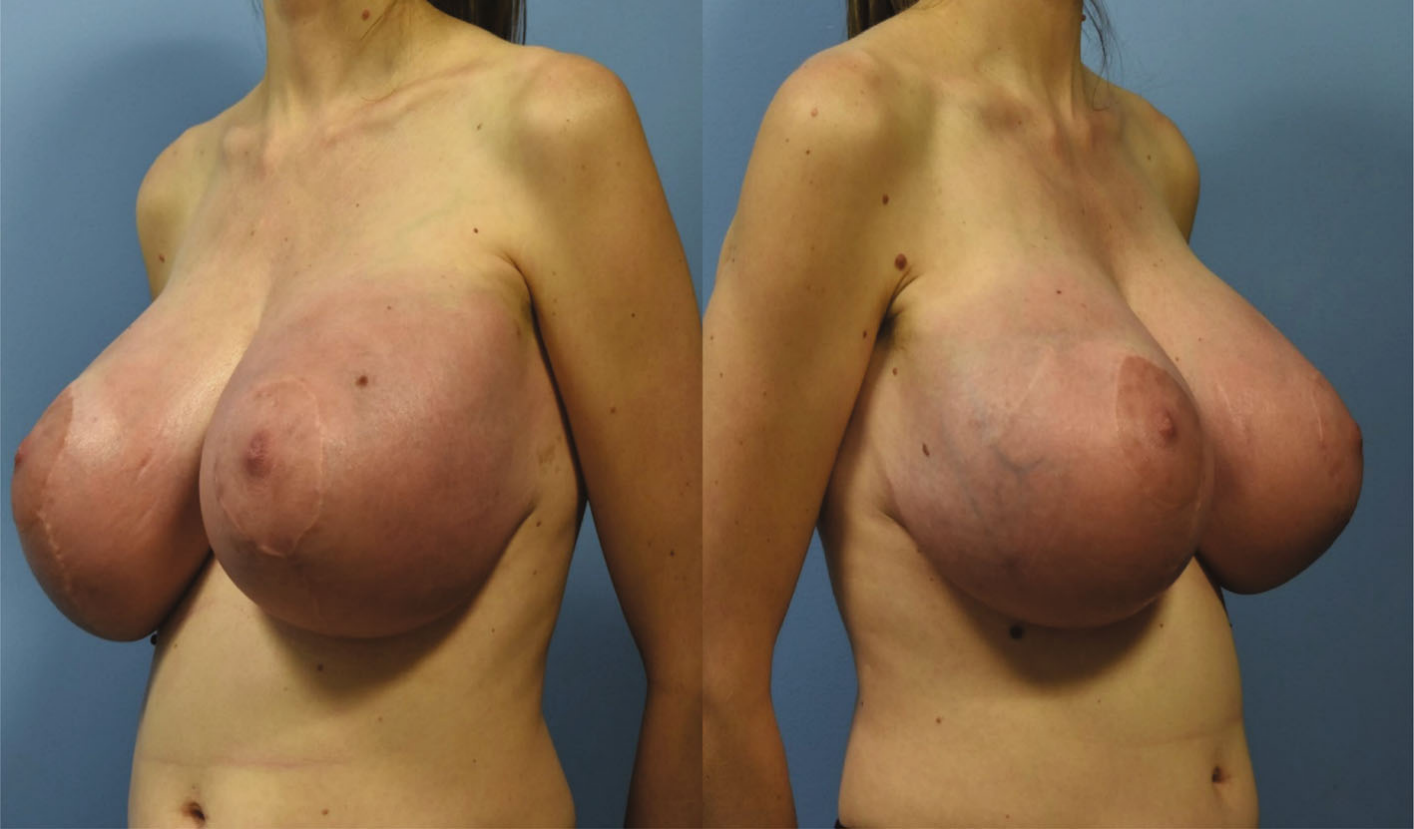
Patient presentation during pregnancy. The patient presented with markedly enlarged breasts and taut darkening skin with a sluggish capillary refill.

Thirteen weeks after conceiving by IVF, the patient reported rapid bilateral breast enlargement. At 16 weeks gestation, she noted shortness of breath attributable to breast weight. By 20 weeks gestation, there was taut darkening skin, moderate erythema, and edema throughout her bilateral breasts, as well as bra strap grooving and tenderness with palpation. The skin had multiple large, visible, subcutaneous venous sinuses. The dependent regions of her breasts, including her nipple areolar complex, had markedly delayed capillary refill. Her bra size had also changed from a B to a G cup (Figure 2). Maternal Fetal Medicine (MFM) consultants concurred with the recommendation for mastectomy. Given her worsening respiratory symptoms, signs of early skin compromise, and breast pain, a bilateral mastectomy procedure was performed. The patient was offered breast reconstruction with tissue expander placement but declined.

### 2.3. Intraoperative Course

Optimal patient positioning for the mastectomy was achieved by placing the patient in a left lateral tilt position with a wedge under her right hip to displace the gravid uterus off the inferior vena cava. When needed, the bed was airplane in either direction to better accommodate bilateral surgical access. The greatest blood loss occurred at the time of skin incision using a scalpel with an immediate 200 cc loss for several minutes. Cautery devices including Bovie and Ligasure (Medtronic, Minneapolis, MN, USA) were not able to seal these massive venous sinuses. The vessels were also too friable for clip placement. Instead, suture ligation of all dilated superficial vessels was serially performed to obtain hemostasis. This was followed by the development of the mastectomy flaps superiorly and inferiorly with Bovie cautery and suture ligation of all prominent deep vessels. The medial flap was then developed with care paid to isolate the perforating branches of the internal thoracic artery at the level of the second rib for suture ligation. Dissection of the lateral flap was most notable for very prominent vessels, especially at the region of the axillary tail that also required suture ligation. The mastectomy specimens weighed 2820 g on the left side and 2995 g on the right side. The estimated total blood loss for the procedure was 300 cc, not accounting for passive blood loss from the removal of her massive breasts. Her preoperative hematocrit (HCT) was 34, and her postoperative HCT was 27.

### 2.4. Postoperative Course

**Figure 3:**
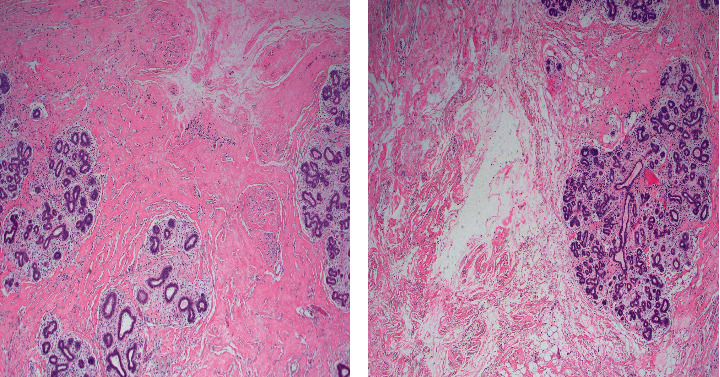
Histological tissue examination of PASH. Marked edema and expansion of the interlobular stroma with areas of stromal hyperplasia, including conventional pseudoangiomatous stroma hyperplasia with clefts and fascicular PASH (a and b). On immunohistochemistry, the stromal cells were estrogen receptor-negative, smooth muscle actin positive, and CD34 positive consistent with myofibroblasts.

While heparin and enoxaparin can be used in pregnancy as they do not cross the placenta, we chose to postpone initiation until postoperatively, due to the concern for increased risk of bleeding and hematoma. Our patient received only preoperative pneumoboots for deep vein thrombosis and enoxaparin postoperatively until she was fully ambulatory. Her pain was minimal and controlled with Tylenol alone, although narcotics and short-term use of indomethacin (up to 48 hours) can be safely added for pain management. The patient was discharged home after 48 hours. The patient had her surgical drains removed at 2 weeks, and no antibiotics were given while her drains were in place. Histological tissue examination showed marked edema and expansion of the interlobular stroma with areas of stromal hyperplasia, including conventional PASH with clefts, and fascicular PASH (Figures 3(a) and 3(b)). In the immediate postoperative period, the patient reported significant improvement in her respiratory symptoms and overall well-being. The rest of her pregnancy course was uncomplicated, and she delivered at term via spontaneous vaginal delivery.

## 3. Discussion

Severe breast enlargement from diffuse PASH combined with gestational hypertrophy poses severe health risks to the mother and fetus. As the literature on PASH in pregnancy is sparse, we present here our multidisciplinary approach to the management of this rare disease.

Gestational breast enlargement is normal, and the increase in volume averages 96 mL [4]. However, in extreme cases, the weight of the breasts causes chest wall compression that interferes with maternal breathing [5]. Maternal lung capacity is further reduced by normal gestational uterine distention that pushes the diaphragm upwards. Breasts become edematous, so skin ulceration and necrosis can occur. Fetal perfusion and fetal growth may be impaired by severe breast enlargement as increasing breast vascularization reroutes blood supply from the fetus to the breasts [6]. Furthermore, as PASH is thought to be promoted by increased progesterone levels [7], accelerated breast enlargement may continue throughout pregnancy, and may not resolve after delivery given normal enlargement for lactation. In our case, alternatives, such as inpatient admission, bed rest, and respiratory support, will not adequately address the patient's symptoms and would increase the risks for thromboembolic complications. Furthermore, it would not have been feasible to keep the patient's *body positioned* in the left lateral lie throughout her pregnancy.

Providers may not recognize these potential comorbidities since gigantomastia from gestational PASH is often considered a *benign* disease. Our patient, for example, focused on monitoring her skin for necrosis but was not aware of potential complications to the pregnancy. Due to the extremely rapid breast growth, expected to continue through the rest of the pregnancy, the already present skin compromise, and the maternal dyspnea, expected to worsen as the uterine fundus enlarged further, our MFM consultants recommended bilateral mastectomies before completing her second trimester of pregnancy. In the second trimester, there is less risk of supine hypotension due to aortocaval compression by the gravid uterus, less risk for reduced uterine blood flow, and minimal risk for preterm labor. Maternal surgeries, especially those that do not enter the abdominal cavity, are usually well tolerated by the fetus, provided gravid patients are positioned appropriately to avoid hypotension, care is taken not to inadvertently lean on the maternal abdomen, and measures are taken to address the possibility of increased blood loss. Early second trimester, after the completion of fetal organogenesis and while the uterine fundus is below the sacral promontory, would be the optimal time for surgical intervention. As long as the precautions mentioned above are taken, intraoperative continuous fetal monitoring, which can be cumbersome and prolong operative time, is not required at previable gestations; a fetal heart rate check before and after surgery is sufficient. MFMs can often reassure surgeons who may overestimate the risks of fetal complications. Interventions that keep mothers healthy usually outweigh theoretical complications.

Surgical challenges to performing the patient's mastectomy result from the dramatic growth of maternal mammary vasculature as hypertrophy proceeds [1]. Increased vascularization and vessel diameter raise the risk of intraoperative bleeding and are more difficult to control once vessels have ruptured. The first mastectomy described for massive hypertrophy of the breasts in pregnancy was in 1958 by Blaydes and Kinnebrew [8]. The patient underwent bilateral mastectomies (5350 and 4000 g) at 6.5 months of pregnancy after spontaneous rupture of a venous sinus resulting in massive hemorrhage and shock. Her mastectomies were through vertical incisions requiring skin grafting, and the patient needed 16 units of blood. Given the risk of excessive bleeding, blood should be crossmatched ahead of time. In our institutional experience, the need for blood transfusion in these surgical cases is rare.

The plastic surgery service was consulted in the preoperative setting to discuss immediate breast reconstruction with tissue expander placement. This notion was supported by successful breast reconstruction in this setting at our institution and also one case report identified in our review of the literature [3]. The patient declined reconstruction to reduce operative time and infection given her strong desire to minimize all potential complications. A plastic surgeon designed a large elliptical incision for her mastectomy given her prior reduction mammoplasty and complex closure scars. It has been 2 years since the patient's delivery and although she is a candidate for autologous breast reconstruction, the patient has been content with her flat closure.

In hindsight, given the patient's rapid interval regrowth of PASH despite her multiple excisions and her plan to get pregnant through assisted reproductive therapies entailing the use of estrogens and progesterone, earlier intervention with mastectomy rather than temporization with sequential reduction mammoplasty would have been preferable. This option had been offered to the patient and declined. Reported PASH recurrence rates after excision vary widely from 0% to 22% [1, 9–14], and the risk of PASH recurrence in pregnancy is not known. To mitigate maternal/fetal health risks and the risk of reoperation, our case suggests that providers should strongly consider upfront bilateral mastectomy rather than breast reduction for patients with severe diffuse PASH who are planning to get pregnant. This strategy is also supported by previous case reports [6, 15].

Given the rarity of PASH resulting in gigantomastia in pregnancy, with a reported incidence of 1 : 100,000 [6], most breast care and obstetrical providers will seldom encounter this clinical scenario in their careers. However, when such a patient does present for care, our review of the literature yielded very limited guidelines. We report this case to share how multidisciplinary management can improve outcomes for the mother and fetus. Our collective recommendation is that pregnant women with suspected PASH should undergo multidisciplinary consultation with a Breast Cancer Surgeon, MFM Obstetrician, and Plastic Surgeon immediately and preferably before the second trimester such that surgical planning can be made promptly. Pregnant women who undergo mastectomies for PASH-induced gigantomastia during their second trimesters will likely recover quickly and do well from an obstetrical perspective, and fetal risks are low.

## Data Availability

We do not have additional data or files. All required information is included in the manuscript.
